# Evaluation of Different Detection and Identification Methods of Intact Tetrahydro‐Methyltestosterone Sulfate Metabolites in Doping Control

**DOI:** 10.1002/dta.70060

**Published:** 2026-03-15

**Authors:** Yiannis S. Angelis, Olga Goula, Polyxeni Kiousi, Panagiotis Sakellariou

**Affiliations:** ^1^ Doping Control Laboratory of Athens Institute of Biosciences and Applications Athens Greece; ^2^ Institute of Biochemistry/Center for Preventive Doping Research German Sport University Cologne Cologne Germany

**Keywords:** 17α‐methyltestosterone, anabolic androgenic steroids, doping control, identification, sulfate metabolites

## Abstract

Tetrahydro sulfate metabolites are well‐established long‐term biomarkers of methyltestosterone use that can be detected intact by LC‐MS/MS. However, their identification using product ion spectra under collision‐induced dissociation conditions in negative ion mode is an analytical challenge under the provisions of the WADA TD2023IDCR. In this study, six out of eight potential tetrahydro‐methyltestosterone sulfate metabolites were microscale‐synthesized to facilitate both the structural elucidation of the detected metabolites and the development of direct identification methods. Their identification was based on their GC‐MS/(MS) analysis after TMS derivatization or LC‐HRMS/(MS) analysis following derivatization of the free 17β‐hydroxy group with carbonyldiimidazole (CDI), producing 17β‐OH‐imidazole carbamate derivatives. The resulting derivatives were detectable in both negative and positive ion modes, enabling their identification through characteristic product ion spectra. Urine samples from two 17α‐methyltestosterone excretion studies were analyzed using these methods, and detection/identification time windows of intact sulfate metabolites were estimated under TD2023IDCR and compared with those obtained from GC‐MS/(MS) analysis of the glucuronide fraction after hydrolysis. Overall, the inclusion of the tetrahydro‐methyltestosterone sulfate metabolites significantly extends the detection time window for methyltestosterone abuse. Still, the established identification time window was similar to, or shorter than, that derived from the glucuronide fraction analysis.

## Graphical abstract

The micro‐scale synthesis of the six out of eight possible tetrahydro‐methyltestosterone sulfate metabolites was performed, and the synthesized materials were used as analytical standards. The analysis of post‐administration sample extracts from methyltestosterone excretion studies against these standards provides further experimental proof for their structural assignment. Additionally, a novel LC/MS identification method was developed after derivatization for the major intact methyltestosterone sulfate metabolite, and the resulting identification time windows were evaluated against well‐established GC‐MS, CP, and ITP methods.

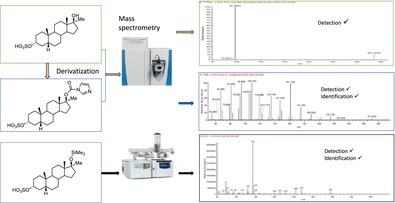

## 1 Introduction

17α‐methyltestosterone (MT) is the prototype of a series of 17α‐alkylated steroids developed to increase the oral bioavailability of anabolic androgenic steroids. It was first synthesized in 1935 [1] and introduced in clinical practice soon afterwards for medical conditions related to low levels of androgens [2]. As MT is both anabolic and androgenic, it can increase muscle mass and strength [3], and has therefore been abused in sport [4] and prohibited by the World Anti‐Doping Agency (WADA) [5]. MT is extensively metabolized in the human body, and its metabolic pathways have been investigated on several occasions [6, 7, 8, 9, 10, 11, 12, 13]. A comprehensive metabolic profile of MT has been presented [11, 12]. The main metabolic pathways include the reduction of the A ring of the steroid skeleton, leading to the tetrahydro‐steroid metabolites, 17α‐methyl‐5α‐androstane‐3α,17β‐diol and 17α‐methyl‐5β‐androstane‐3α,17β‐diol. Further conjugation of these phase I metabolites with glucuronic and/or sulfuric acid leads to target analytes for sports drug testing analysis.

**FIGURE 1:**
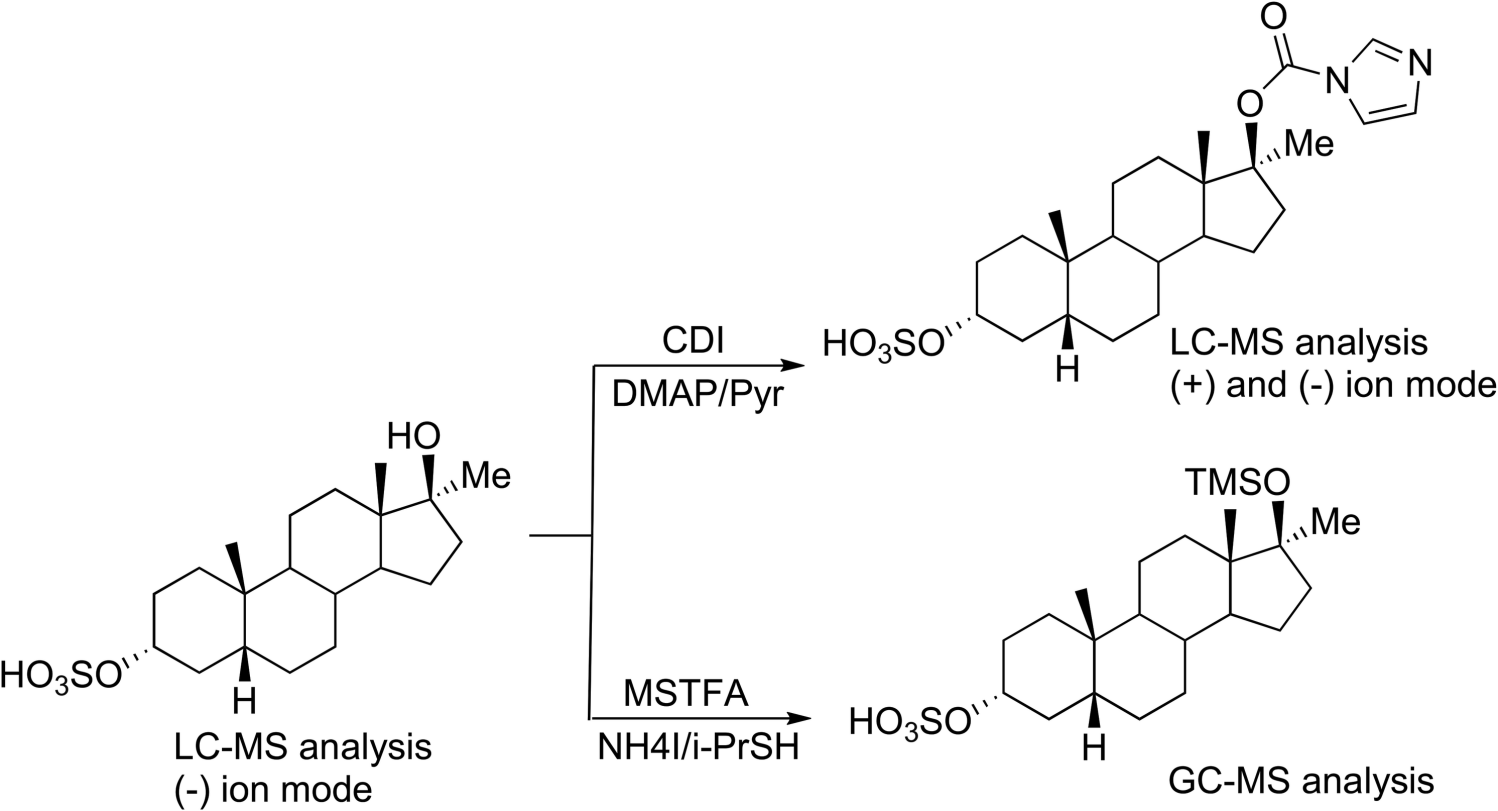
Structure of a representative tetrahydro‐methyltestosterone sulfate metabolite and its derivatives designed for use in different analytical techniques.

**FIGURE 2:**
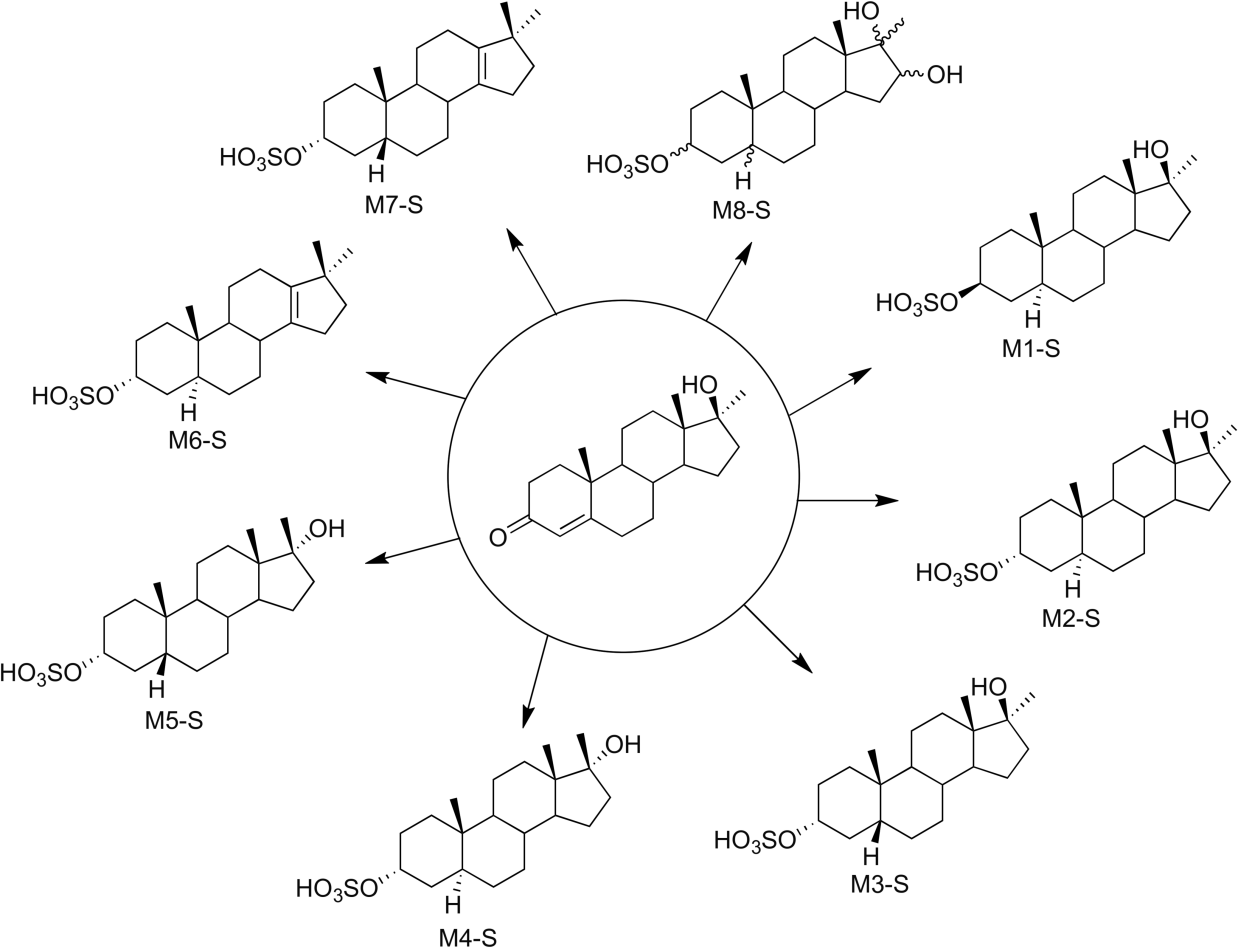
Investigated tetrahydro‐methyltestosterone sulfate metabolites. M1‐S corresponds to 17α‐methyl‐5α‐androstane‐3β,17β‐diol‐3β‐sulfate, M2‐S to 17α‐methyl‐5α‐androstane‐3α,17β‐diol‐3α‐sulfate, M3‐S to 17α‐methyl‐5β‐androstane‐3α,17β‐diol‐3α‐sulfate, M4‐S to 17β‐methyl‐5α‐androstane‐3α,17α‐diol‐3α‐sulfate, M5‐S to 17β‐methyl‐5β‐androstane‐3α,17α‐diol‐3α‐sulfate, M6‐S to 17,17‐dimethyl‐18‐nor‐5α‐androst‐13‐ene‐3α‐sulfate, M7‐S to 17,17‐dimethyl‐18‐nor‐5β‐androst‐13‐ene‐3α‐sulfate, and M8‐S to 16z‐hydroxy‐tetrahydro‐methyltestosterone sulfate. Please note that when the glucuronide analogs of these metabolites are referred to in the following sections of the manuscript, the same coding is retained, with the extension of the metabolite code changed from‐S to ‐G, (e.g., M3‐G).

WADA‐accredited doping control laboratories may monitor methyltestosterone in human urine by the detection of its main metabolites, 17α‐methyl‐5β‐androstane‐3α,17β‐diol and 17α‐methyl‐5α‐androstane‐3α,17β‐diol using gas chromatography‐(tandem) mass spectrometry (GC‐MS/(MS)), after hydrolysis with β‐glucuronidase of their glucuronide conjugates and subsequent per‐trimethylsilyl (TMS) derivatization. However, analysis of the intact phase II metabolites by liquid chromatography–tandem mass spectrometry (LC‐MS/MS) revealed the presence of epimeric tetrahydro‐methyltestosterone metabolites and especially the 3α‐sulfate derivative of 17β‐methyl‐5α‐androstane‐3α,17α‐diol, which substantially prolongs the detection time window for methyltestosterone [8, 11]. These epimeric metabolites are produced through epimerization of the 17β‐sulfate group as part of the phase II metabolism [14]. Structural assignment for these important metabolites was made indirectly through sulfate deconjugation after GC‐MS/(MS) analysis [8]. Recently, the synthesis of the major tetrahydro‐methyltestosterone metabolite was reported [15] corresponding to 17α‐methyl‐5β‐androstane‐3α,17β‐diol‐3α‐sulfate (Figure 1, coded as M3‐S herein) and its direct analysis against post‐administration samples confirmed the tentative structure already proposed by Gomez et al. [8]. The same authors also reported that a minor metabolite, indirectly characterized as the 3α‐sulfate derivative of the epimeric 17β‐methyl‐5α‐androstane‐3α,17α‐diol (Figure 2, coded as M4‐S herein) [8], shows the highest retrospectivity [8, 12]. Yet, direct experimental proof that would confirm the correct assignment of its structure is still lacking. In this study, microscale synthesis of six out of eight possible tetrahydro‐methyltestosterone sulfate metabolites and two out of four possible 18‐nor‐17,17‐dimethyl‐Δ_13_‐androsten‐3z‐sulfates was performed, and the synthesized material was used as standards for the unambiguous structural assignment of tetrahydro‐methyltestosterone sulfate metabolites extracted from post‐administration urine samples.

Ideally, the prolonged detection time windows offered by the monitoring of the tetrahydro‐methyltestosterone sulfate epimers should be accompanied by equal or prolonged identification time windows under the provisions of WADA's TD2023IDCR. However, the identification of these metabolites, in the case of suspicious samples, in negative ion mode is hindered by their poor product ion spectra under collision‐induced dissociation (CID) conditions, which contain product ions related exclusively to the sulfate moiety, providing limited structural information [8, 11, 16]. The development of a direct method for their identification as intact sulfate conjugates remains an analytical challenge. In this context, GC‐MS analysis of intact sulfate metabolites of MT, in which the desulfated artifacts of their TMS derivatives are detected (Figure 1), has been reported [17, 18] and, to the best of our knowledge, represents the only available identification approach in which confirmation of intact sulfate conjugates is based on ion transitions related to the steroid moiety and not to the conjugation group.

An alternative strategy has been proposed for the identification of oxo‐steroids' sulfate metabolites, where the product ion spectra can be substantially altered after derivatization with Girard's reagent T [16, 19]. This derivatization enables their identification by liquid chromatography‐high resolution (tandem) mass spectrometry (LC‐HRMS/(MS)) in positive ion mode. In this study, the extension of this derivatization strategy to the identification of intact sulfated fully saturated metabolites of MT, targeting the free 17‐hydroxyl group of the steroids as the derivatization site, using 1,1′‐carbonyldiimidazole (CDI) as the derivatization reagent, was investigated. The tetrahydro‐methyltestosterone sulfate standards synthesized in the frame of this study were used to develop an LC‐MS method based on CDI derivatization, in which samples were analyzed in both positive and negative ion modes. Advantages and pitfalls of this derivatization methodology were evaluated, and the developed method was applied to post‐administration sample extracts from two different administration studies. For the assessment of this identification strategy's efficiency, the results were compared with those derived from the identification of the same metabolites in the same samples analyzed as de‐sulfated artifacts by GC‐MS/(MS) [11], as well as with results obtained from the typical analysis of the glucuronide fraction by GC‐MS/(MS) after deconjugation with β‐glucuronidase from *E. coli* [12]. The metabolites that were investigated are presented in Figure 2 alongside their assigned codes.

## 2 Experimental

### 2.1 Chemicals and Reagents

Androsterone sulfate sodium salt, etiocholanolone sulfate sodium salt and epiandrosterone sulfate sodium salt were purchased from LGC standards (Middlesex, UK). Androsterone, etiocholanolone, 17α‐methyl‐5β‐androstane‐3α,17β‐diol, 17α‐methyl‐5α‐androstane‐3α,17β‐diol, 17β‐methyl‐5α‐androstane‐3α,17β‐diol and *d*4–19‐norandrosterone glucuronide were purchased from the National Measurement Institute (NMI, Canberra, Australia). Epiandrosterone was purchased from Steraloids Inc. (Newport, USA). Methyltestosterone, ammonium iodide (NH_4_I), 2‐propanethiol (2‐PrSH), methylmagnesium bromide 3 M in diethyl ether, sulfur trioxide pyridine complex, dimethylformamide (DMF), 1,4‐dioxane, carbonyldiimidazole (CDI), sodium hydroxide (NaOH) and 4‐Dimethylaminopyridine (DMAP) were of analytical grade and were obtained from Sigma‐Aldrich (Steinheim, Germany). Diethyl ether, n‐pentane, ethyl acetate, dichloromethane, and methanol (MeOH) were of analytical grade and were obtained from Labscan (Dublin, Ireland). *β*‐glucuronidase from *E. coli* was obtained from Roche Diagnostics GmbH (Mannheim, Germany). Dipotassium hydrogen phosphate (K_2_HPO_4_), potassium dihydrogen phosphate (KH_2_PO_4_), disodium carbonate (Na_2_CO_3_), sodium hydrogen carbonate (NaHCO_3_), anhydrous sodium sulfate (Na_2_SO_4_), hydrochloric acid 37% (HCl), glacial acetic acid, and acetonitrile (ACN) were of analytical grade and were purchased from Panreac (Barcelona, Spain). N‐methyl‐N‐trimethylsilyltrifluoroacetamide (MSTFA) was purchased from Chemische Fabrik Karl Bücher GmbH (Waldstetten, Germany). Milli‐Q water was obtained using a Milli‐Q purification system (Millipore, Billerica, MA, USA). Solid‐phase extraction (SPE) C18 cartridges (500 mg, 6 mL) were purchased from Agilent Technologies (Santa Clara, USA).

### 2.2 Synthesis of Analytical Standards

The synthesis of analytical standards of tetrahydro‐methyltestosterone diols conjugated in the 3‐hydroxy group of the steroid A‐ring, with well‐defined stereochemistry at 3, 5, and 17 positions of the steroid skeleton, was performed via Grignard reaction of methylmagnesium bromide using the corresponding 17‐ketosteroids as substrates [20, 21]. Specifically, 100 μL of androsterone sulfate, etiocholanolone sulfate, and epiandrosterone sulfate solutions (100 ng/mL in MeOH) were transferred to test tubes, evaporated to dryness, and reconstituted in 0.5 mL of 1,4 dioxane. Then, 0.2 mL of methylmagnesium bromide 3 M in diethyl ether was added dropwise with stirring at room temperature. Upon completion of the addition, 0.2 mL of NaOH 0.1 N was immediately added dropwise with stirring. After hydrogen evolution ceased, 5 mL of water was added, and the resulting white emulsion was acidified with 0.2 mL of glacial acetic acid. The reaction mixture was extracted three times with n‐pentane, and the extracts were discarded. The reaction products were isolated by SPE on preconditioned C18 cartridges after elution with 5 mL of MeOH.

For the synthesis of 17,17‐dimethyl‐18‐nor‐Δ_13_‐tetrahydro‐methyltestosterone analytical standards, the above‐mentioned reaction products were evaporated to dryness and reconstituted in 0.2 mL of ACN. Then, 0.3 mL of 3 M HCl (aqueous) was added, and the reaction mixture was heated at 80°C for 30 min. The reaction products were isolated by SPE on preconditioned C18 cartridges after elution with 5 mL of MeOH.

### 2.3 Human Administration Study

Urine samples were obtained after the single‐dose administration of Teston to two healthy male human volunteers (Series I: Caucasian, 40 years old, 90 kg, 1 capsule × 25 mg of methyltestosterone; Series II: Caucasian, 53 years old, 101 kg, 1/2 capsule × 25 mg of methyltestosterone). Urine samples were collected before (0 h) and up to 403 h after administration for Series I. In Series II, samples were collected before (0 h) and up to 493 h. The exact time intervals (h) were as follows. Series I: 0, 1, 8.5, 17.5, 23, 26, 41.5, 47.5, 53, 65.5, 72, 103, 127, 144, 170, 194, 215, 313, 360, and 403. Series II: 0, 1, 3.5, 7, 14.5, 19, 5 24.5, 36, 5, 41.5, 48, 63, 66, 89.5, 115.5, 138.5, 201, 226, 248, 273, 294, 319, 345, 362, 369, 385, 390, 413, 436, 472, and 493. All collected samples were kept frozen at −20°C until analysis. The study was approved by the Bioethics Committee of the National Centre for Scientific Research (NCSR) “Demokritos”, Athens, Greece (authorization code: 240/2022‐1612/92‐19‐12‐2022).

### 2.4 Sample Preparation

#### 2.4.1 Sample Preparation Procedure for the Detection and Identification of Methyltestosterone Metabolites (Free and Glucuronide)

For the extraction of the free and glucuronide steroids, *d*4–19‐norandrosterone glucuronide (20 μL of a 1.25 μg/mL solution) was added to 3 mL urine samples as an internal standard (ISTD) in glass test tubes. The pH was adjusted to 7.0 using phosphate buffer 1 M, and urine samples were incubated for 1.5 h at 55°C after the addition of 100 μL β‐glucuronidase from *E. coli* . Following hydrolysis, urine pH was adjusted to 9.5 with NaHCO_3_:Na_2_CO_3_ (10:1, w/w) solid buffer, and free steroids were extracted twice by shaking for 10 min with 5 mL *n*‐pentane. In each extraction step, samples were centrifuged, the organic layer was transferred to glass tubes, and evaporated to dryness under nitrogen at 50°C. Per‐TMS derivatives were prepared by adding 50 μL MSTFA/NH_4_I/2‐PrSH (1000:4:3, v/w/v) to the dry residue. The mixture was incubated at 80°C for 30 min. After cooling, samples were transferred to vials equipped with inserts, and 2 μL of the solution was then injected directly into GC‐MS/(MS).

#### 2.4.2 Sample Preparation for the Extraction of the Sulfate Metabolites of Methyltestosterone

The remaining urine residues from step 2.4.1 were extracted twice with 5 mL of ethyl acetate by shaking for 10 min. Samples were centrifuged in each step, and the organic layer was transferred to the glass tubes and evaporated to dryness under nitrogen at 60°C.

##### 2.4.2.1 Intact Sulfate Metabolites

For the analysis of the underivatized intact sulfate metabolites by LC‐HRMS/(MS), the samples were reconstituted with 120 μL ACN/H_2_0 (20/80, v/v). The reconstituted samples were transferred to Eppendorf tubes and centrifuged for 15 min at 10,000 rpm. Then, 100 μL of the supernatant was transferred to vials with inserts. A 10 μL was injected into the instrument without further purification.

##### 2.4.2.2 CDI Derivatives of Intact Sulfate Metabolites

For CDI derivatives, 100 μL of a freshly prepared CDI solution (0.1 M in dry dichloromethane), containing 0.5% (w/v) DMAP and 0.5% (v/v) pyridine, was added to the dry residues of the samples. The derivatization mixture was incubated at 70°C for 30 min. After cooling, samples were reconstituted in 80 μL of ACN and transferred to vials equipped with inserts. A 10 μL of the solution was then injected directly into the LC‐HRMS/(MS).

##### 2.4.2.3 TMS Derivatives of Intact Sulfate Metabolites

TMS‐derivatization for GC‐MS/(MS) was performed by adding 50 μL MSTFA/NH_4_I/2‐PrSH (1000:4:3, v/w/v) to the dry residue. The mixture was incubated at 80°C for 30 min. After cooling, samples were transferred to vials equipped with inserts, and 2 μL of the solution was then injected directly into GC‐MS/(MS).

### 2.5 Instrumentation

#### 2.5.1 LC‐HRMS/(MS)

A Dionex UHPLC system (Thermo Scientific, Bremen, Germany) was used for chromatographic separation. The system consisted of a vacuum degasser, a high‐pressure binary pump, an autosampler with a temperature‐controlled sample tray set at 15°C, and a column oven set at 30°C. Chromatographic separation was performed at 30°C using a Zorbax Eclipse Plus C18 column (100 × 2.1 mm i.d., 1.8 μm particle size; Agilent Technologies). The mobile phase consisted of 5 mM ammonium formate in 0.02% formic acid (Solvent A) and a mixture of acetonitrile: water (90:10, v/v) containing 5 mM ammonium formate and 0.02% formic acid (Solvent B). For the efficient resolution of the different isomeric analytes of intact tetrahydro‐methyltestosterone sulfate metabolites, a gradient incorporating a double isocratic elution program was employed, with Solvent B starting at 30% for 13 min, increasing to 41% at 13.5 min, where it was held until 21 min, then increased to 60% at 26 min and to 100% at 28 min, where it remained until 31 min. Solvent B was returned to 30% at 31.5 min and equilibrated for 3.5 min. The flow rate was 0.2 mL min^−1^ and the total chromatographic time was 35 min. The injection volume was 10 μL. The diverter valve was programmed to send LC eluent to waste for the first 5 min. The chromatographic program employed for the analysis of the CDI derivatized intact tetrahydro‐methyltestosterone sulfate metabolites, started with Solvent B at 65%, increasing to 70% at 4 min, then to 80% at 8 min and to 100% at 11 min, where it was held until 16 min. The gradient was returned to 65% at 16.5 min and equilibrated for 3.5 min. The flow rate was again 0.2 mL min^−1^ and the total chromatographic time was 20 min. The injection volume was 10 μL. The diverter valve was programmed to send LC eluent to waste for the first 5 min.

A QExactive benchtop Orbitrap‐based mass spectrometer (Thermo Scientific, Bremen, Germany) operated in negative ion mode and equipped with a heated electrospray ionization (HESI) source was used as a detector. Source parameters were; sheath gas (nitrogen) flow rate, auxiliary gas (nitrogen) flow rate, and sweep gas flow rate; 40, 10, and 1 arbitrary units, respectively; capillary temperature; 300°C; electrospray ionization (ESI) heater temperature; 30°C; and spray voltage; 4.0 kV. Full scan experiments were conducted with a range of *m/z* 50–750 at a resolution of 17,500 full width at half maximum (FWHM) and injection time of 100 ms. Additional MS/MS experiments were conducted at a resolution of 17,500 FWHM and injection time of 100 ms in parallel reaction monitoring (PRM) mode. The automatic gain control (AGC) was set at 3e6 ions. For the detection of intact sulfate metabolites, the samples were analyzed in negative ion mode. The precursor ions at *m/z* 385.2049 and 367.1943 were isolated with an isolation window of *m/z* = 1. For the detection of CDI derivatives of intact tetrahydro‐methyltestosterone sulfate metabolites, the samples were analyzed in two subsequent runs: the first in negative ion mode where precursor ions at *m/z* 479.2216 and 367.1943 were isolated with an isolation window of *m/z* = 1 and the second run in positive ion mode where precursor ions at *m/z* 271.2426 were isolated with an isolation window of *m/z* = 1. Mass calibration of the Orbitrap was evaluated in both positive and negative modes weekly, and external calibration was performed prior to use following the manufacturer's protocol.

#### 2.5.2 GC‐MS/(MS)

A Trace 1610 GC system (Thermo Scientific, Bremen, Germany), combined with a TSQ9610 (Thermo Scientific, Bremen, Germany), triple quadrupole mass selective detector, equipped with an advanced electron ion source, was used. The system was equipped with SGE BPX5 column (30 m length, 0.25 mm i.d., 0.1 μm film thickness). Helium was used as carrier gas at a constant flow rate of 1.65 mL min^−1^. A 2 μL of the sample was injected in split mode (10:1). The injection port and the interface temperatures were set at 280°C. The initial oven temperature was set at 160°C, ramped at 10°C min^−1^ to 200°C, then at 2°C to 200°C, at 6°C min^−1^ to 263°C and finally at 50°C min^−1^ to 310°C where it was held for 1.6 min. Argon was used as collision gas at a pressure of 4 bar. The mass spectrometer was operated in multiple reaction monitoring (MRM) mode.

## 3 Results and Discussion

### 3.1 Microscale Synthesis of MT Sulfate Metabolites

Analysis of the free and glucuronide fractions of post‐administration samples from MT excretion studies [12] revealed the presence of the glucuronide analogs of M4‐G and M5‐G metabolites, as well as their dehydrated Wagner‐Meerwein rearrangement products M6‐G and M7‐G, in addition to the well‐known glucuronide analogs of M2‐G and M3‐G metabolites (Figure 2). The sulfate analogues of these epimeric tetrahydro‐methyltestosterone metabolites have been reported and are considered long‐term metabolites for the monitoring of methyltestosterone abuse in the context of sports drug testing [8]. Based on the long‐term detectability of these metabolites [8] and the reaction mechanism that simultaneously results in the epimerization of the 17‐hydroxy‐17methyl groups of steroids and the Wagner‐Meerwein rearrangement products, leading to 17,17‐dimethyl‐18‐nor‐androst‐13‐ene metabolites [14], the targeted investigation of post‐administration samples was further conducted on the sulfate fraction for these metabolites. For this reason, microscale synthesis of tetrahydro‐methyltestosterone sulfate metabolites was performed through a Grignard reaction of androsterone sulfate, etiocholanolone sulfate, and epiandrosterone sulfate with methylmagnesium bromide using a previously developed protocol [19, 20]. The reaction proceeded in all cases with high selectivity, yielding over 95% for the 17α‐methyl isomer, while the remaining product was the 17β‐methyl steroid. It is worth noting that when the reaction was performed on a larger scale, where methylmagnesium bromide was added under carefully controlled anhydrous conditions at low temperature, and the reaction progress was monitored by thin layer chromatography (TLC), the reaction products recovered were solely the free forms of the corresponding 17‐methylated steroids. The synthesis of the desired products was achieved by the fast addition of the Grignard reagent at room temperature, which was immediately quenched by the dropwise addition of a 0.1 M NaOH solution, simulating titration conditions. Under these conditions, both free and sulfate‐conjugated forms of 17‐methylated‐androstane diols and small quantities of unreacted keto‐steroids were recovered. Free steroids were extracted with n‐pentane and discarded, and the reaction mixture was used without further purification. The produced 17ξ‐methyl‐tetrahydro‐methyltestosterone‐3z‐sulfate mixtures were analyzed by LC‐HRMS in negative ion mode. In all cases, their full scan mass spectra consisted of a molecular ion at *m/z* 385.2059 with mass accuracy better than 2 ppm. The product ion mass spectra acquired in negative ion mode were identical for all potential synthesized MT metabolites under CID conditions at the selected collision energy and contained only two fragment ions at *m/z* 96.9588 and *m/z* 79.9561, both related to the sulfate moiety in accordance with previous findings [8, 15]. Their retention times (RT), along with their observed molecular ions and experimental mass accuracies, are presented in Table 1.

**TABLE 1:** Investigated intact tetrahydro‐MT sulfate metabolites by LC‐HRMS/(MS).

Substance	RT (min)	[M‐H]^−^ (*m/z*)	Mass accuracy (ppm)
Μ1‐S	6.93	385.2056	−0.78
Μ2‐S	8.56	385.2056	−0.52
M3‐S	9.40	385.2054	−1.30
M4‐S	20.70	385.2056	−0.78
M5‐S	21.30	385.2058	−0.26
M6‐S	29.63	367.1949	−1.36
M7‐S	29.68	367.1952	−0.54

**FIGURE 3:**
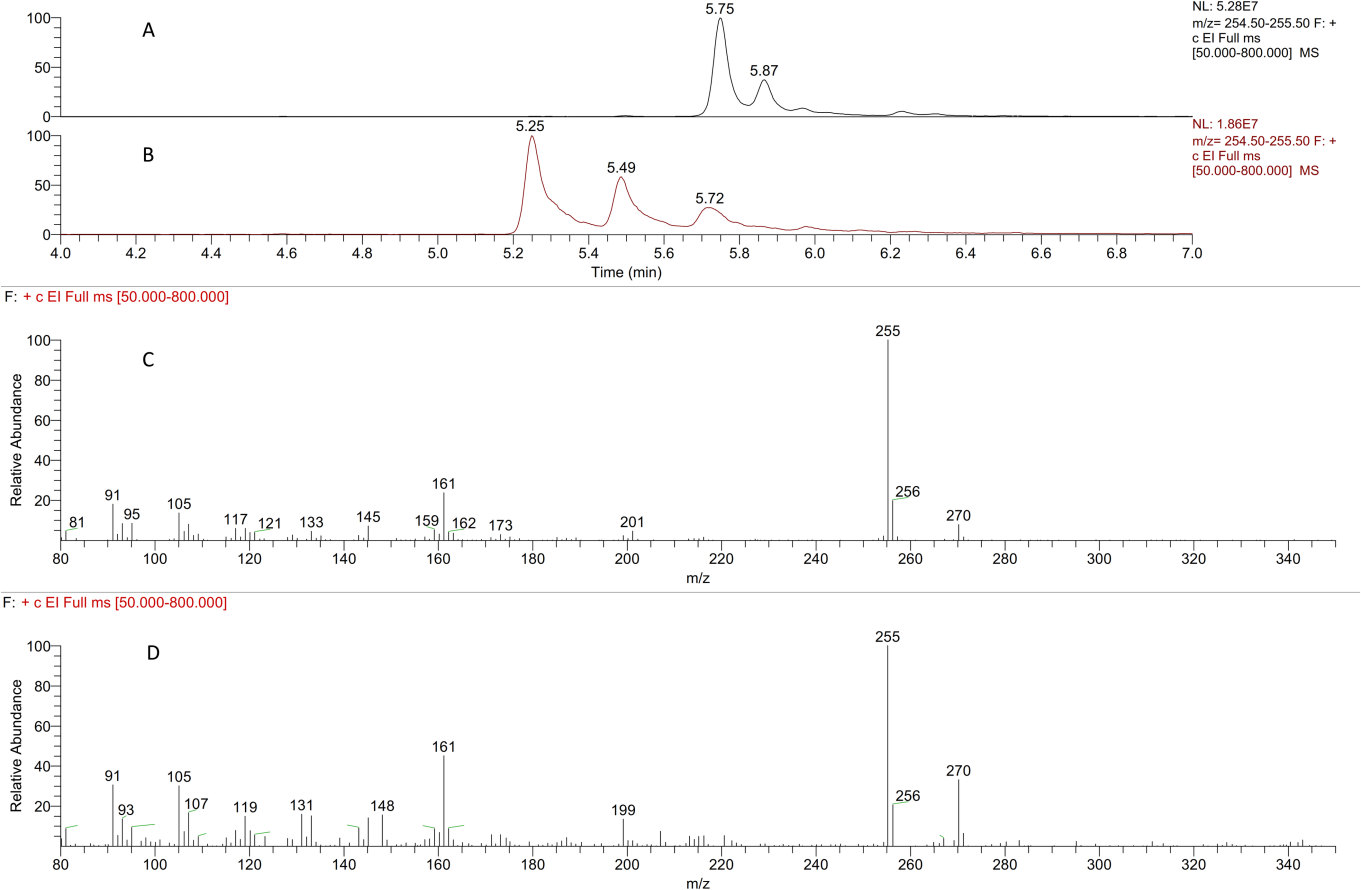
Extracted ion chromatograms at *m/z* 255 of the synthesized (A) M6‐S and (B) M7‐S and background‐subtracted full scan ion spectra of the synthesized (C) M6‐S at 5.75 min and (D) M7‐S at 5.25 min after GC‐MS analysis.

Wagner–Meerwein rearrangement products M6‐S and M7‐S were produced by acidic treatment of the above compounds as described in Section 2.2. Their LC‐HRMS/(MS) chromatographic retention times, their accurate masses (Table 1), and their product ion spectra (data not shown) were almost identical and consistent with their structures. Additionally, their analysis as de‐sulfated artifacts by GC‐MS/(MS) produced different numbers of chromatographic peaks, which were indicative of their structures and were in accordance with their non‐dehydrated analogs [11, 22] as presented in Figure 3.

### 3.2 Structure Elucidation of Intact MT Sulfate Metabolites

**FIGURE 4:**
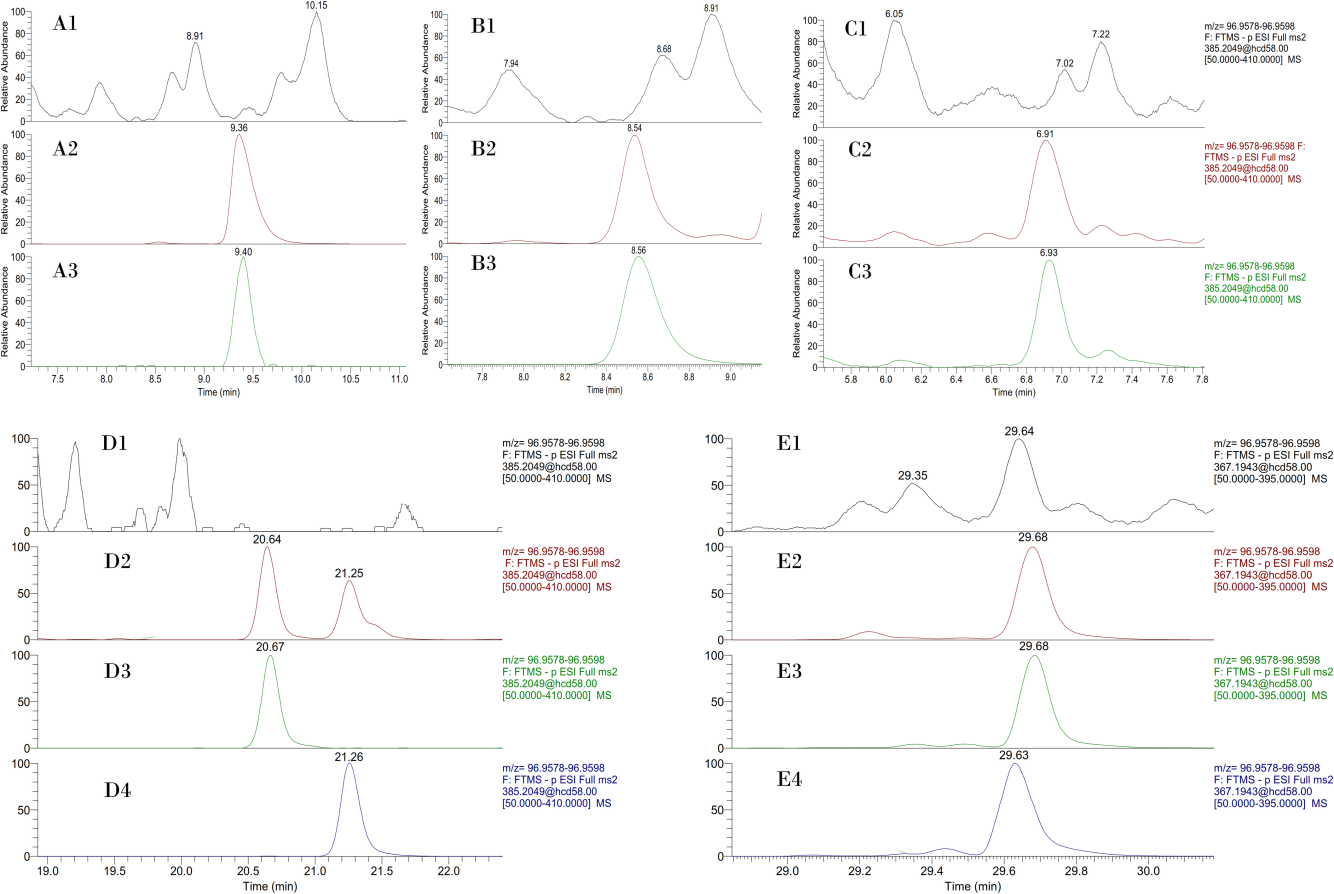
Representative extracted ion chromatograms of: (1) a pre‐administration sample, (2) a 24 h post‐administration sample, (3) a synthetic standard, (4) an isomeric synthetic standard, where relevant after LC‐HRMS/(MS) analysis of intact MT sulfate metabolites. Ion chromatograms correspond to: (A) M3‐S, (B) M2‐S, (C) M1‐S, (D) M4‐S and M5‐S (D3 and D4, respectively) and (E) M6‐S and M7‐S (E3 and E4, respectively).

All samples from the two different excretion studies were analyzed against the synthesized suspected MT intact sulfate metabolites by LC‐HRMS/(MS) in full scan and PRM negative ion modes. The results obtained confirmed previously published results [8, 15] and provided direct experimental proof for the structural assignment of additional MT sulfate metabolites. Representative extracted ion chromatograms of a typical post‐administration sample containing the metabolites of interest in high amounts are presented in Figure 4.

In more detail, M3‐S was detected as the most abundant metabolite in the sulfate fraction in both metabolic studies, consistent with previous results [8, 15] and findings in the glucuronide fraction [12] (Figure 4A). Its 5a analog, M2‐S, was also detected in the post‐administration samples from both excretion studies, but in much lower amounts (Figure 4B). Its 3β‐counterpart, M1‐S was also detected in low amounts (Figure 4C). To the best of our knowledge, the detection of this metabolite in the sulfate fraction of MT is presented for the first time.

The epimeric metabolites M4‐S and M5‐S were also detected (Figure 4D), up to the final collected sample in both excretion studies, in accordance with findings of Gomez et al. [8]. However, the signal‐to‐noise ratio was significantly higher for the first one. Additionally, metabolites M6‐S or M7‐S were also detected (Figure 4E). However, the presence of minor endogenous interferences and the insufficient chromatographic resolution between 5α and 5β metabolites did not allow the unambiguous assignment of the excreted metabolites solely by LC‐MS/HRMS analysis, as co‐injection experiments resulted in a single chromatographic peak in both cases. The structural assignment of these metabolites was therefore further based on the GC‐MS/(MS) analysis of their de‐sulfated artifacts, as presented in Figure 3.

### 3.3 LC‐HRMS/(MS) Analysis of CDI Derivatives of Intact MT Sulphate Metabolites

Chemical derivatization in the case of tetrahydro‐methyltestosterone sulfate metabolites can only be applied to the free 17‐hydroxyl group of the steroid, which is a stereochemically hindered position [23, 24]. A modification of the derivatization protocol applied to hydroxylated free steroids with CDI, which converts hydroxyl groups to imidazole carbamate derivatives (Figure 1), was adopted [25, 26, 27]. More specifically, the efficiency of the CDI derivatization procedure, in the case of 17‐methylated steroids, depends on the conditions under which derivatization is carried out. Hence, while the 17α‐methyl‐5β‐androstane‐3α,17β‐diol could be detected as mono‐CDI derivative, the bis‐CDI derivative could be recovered in a yield higher than 90%, using a small volume of pyridine (0,5% v/v) and DMAP (0,5% w/v) in a CDI solution prepared in a light solvent like dichloromethane, after incubation at 70°C for 30 min. Dichloromethane was allowed to evaporate during derivatization, and the use of pyridine was critical to prevent the dehydration of the 17β‐hydroxyl group, which occurs at higher derivatization temperatures (data not shown). These specific conditions were applied for the derivatization of intact sulfate conjugates of tetrahydro‐MT‐metabolites.

**FIGURE 5:**
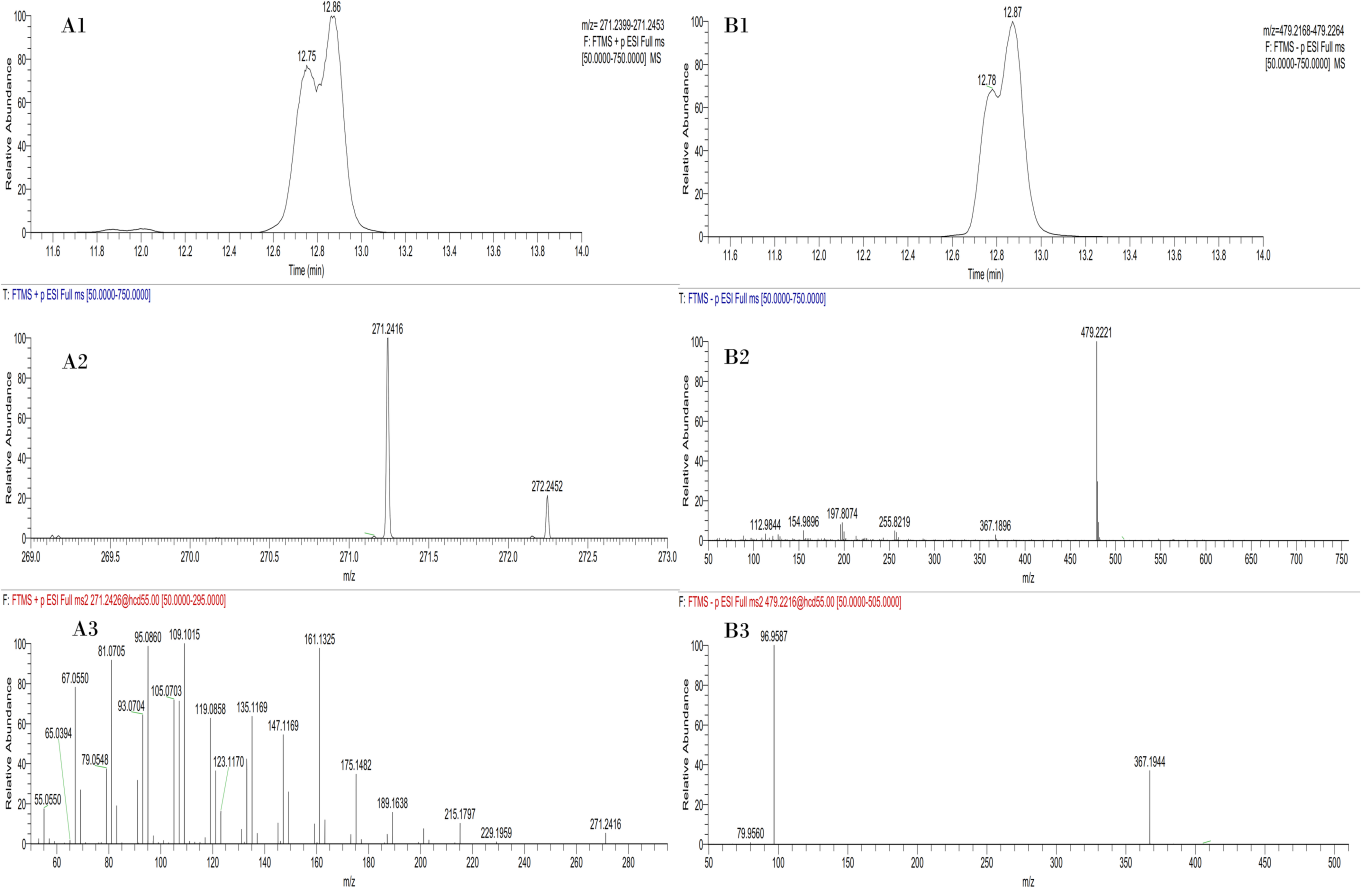
(A1) Full scan extracted ion chromatograms of a standard mixture of Μ2‐S and Μ3‐S at *m/z* 271.2426 in positive ion mode analyzed as imidazole carbamate derivatives by LC‐HRMS/(MS). (A2) Partial full scan spectrum of Μ2‐S and Μ3‐S in positive ion mode. (A3) Product ion spectrum of Μ3‐S in positive ion mode. (B1) Full scan extracted ion chromatograms of a standard mixture of Μ2‐S and Μ3‐S at *m/z* 479.2216 in negative ion mode analyzed as imidazole carbamate derivatives by LC‐HRMS/(MS). (B2) Full scan spectrum of Μ2‐S and Μ3‐S in negative ion mode. (B3) Product ion spectrum of Μ2‐S and Μ3‐S in negative ion mode.

The imidazole carbamate derivatives of the intact M2‐S and M3‐S exhibit identical behavior after LC‐HRMS/(MS) analysis. After full scan analysis in positive ion mode, the only detected signals were for the ion at *m/z* 271.2426, which corresponds to the neat steroid moiety, where both the derivatization group and the sulfate moiety have been eliminated during ESI, as presented in Figure 5A. The product ion spectrum obtained from *m/z* 271.2426 as the precursor ion is typical for steroids [28, 29].

In negative ion mode, the imidazole carbamate derivatives of M2‐S and M3‐S produce an abundant molecular ion at *m/z* 479.2216, along with a secondary ion at *m/z* 367.1943, which corresponds to [M‐H‐Im‐COOH], although to a lesser extent. The product ion spectrum in negative ion mode yields two product ions, one at *m/z* 367.1944, corresponding to [M‐H‐Im‐COOH], which retains the sulfate fraction in the steroid backbone, and a second ion at *m/z* 96.9587, corresponding to the sulfate moiety, characteristic of sulfate metabolites. Full scan and product ion spectra of the imidazole carbamate derivatives of Μ2‐S and Μ3‐S in negative ion mode are presented in Figure 5B.

**FIGURE 6:**
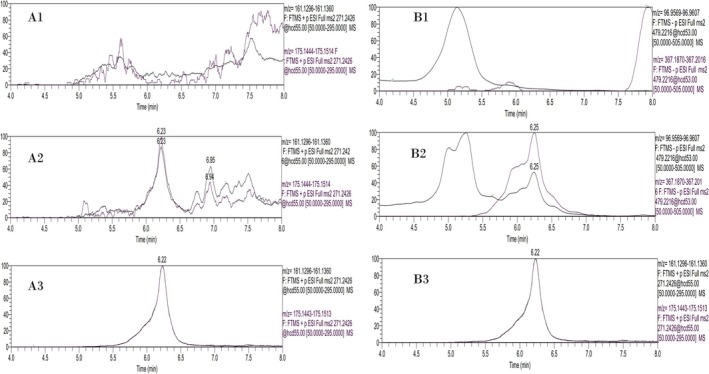
Representative extracted ion chromatograms of Μ3‐S for the ion transitions at *m/z* 271.2426 > 161.1328 and *m/z* 271.2426 > 175.1479 of a (A1) blank sample, (A2) last confirmed sample 9 days post‐administration, and (A3) spiked sample with Μ3‐S in positive ion mode analyzed as imidazole carbamate derivatives by LC‐HRMS/(MS). Extracted ion chromatograms of Μ3‐S for the ion transitions at *m/z* 479.2216 > 367.1943 and *m/z* 479.2216 > 96.9588 of a (B1) blank sample, (B2) last confirmed sample 9 days post‐administration, and (B3) spiked sample with Μ3‐S in negative ion mode analyzed as imidazole carbamate derivatives by LC‐HRMS/(MS).

Unfortunately, no chromatographic peak could be obtained for M4‐S or M5‐S, which exhibited the highest retrospectivity in the analysis of the underivatized post‐administration samples, as presented in Section 3.2. An even higher steric hindrance for the epimeric 17β‐methyl‐17α‐hydroxyl group of the steroids could explain this failure. Nevertheless, the CDI derivatization protocol was further applied to the same extracts of MT post‐administration urine samples described in Section 3.2. Based on (a) the failure to detect the CDI derivatives of M4‐S and M5‐S, (b) the poor chromatographic resolution of M2‐S and M3‐S imidazole carbamate derivatives (Figure 5), and (c) the previous knowledge of the prevalence of the M3‐S among the 5α and 5β isomers [15], a faster chromatographic method was adopted, as described in Section 2.5.1. Samples were analyzed by LC‐HRMS/(MS) in both positive and negative ion mode, and chromatographic and mass spectrometric data were evaluated according to TD2023IDCR criteria. Representative extracted ion chromatograms are presented in Figure 6. Additionally, 20 different blank samples were analyzed with the same method in order to assess its selectivity, and the method proved to be 100% selective (data not shown).

### 3.4 GC‐MS/(MS) Analysis of TMS Derivatives of Intact MT Sulfate Metabolites

**FIGURE 7:**
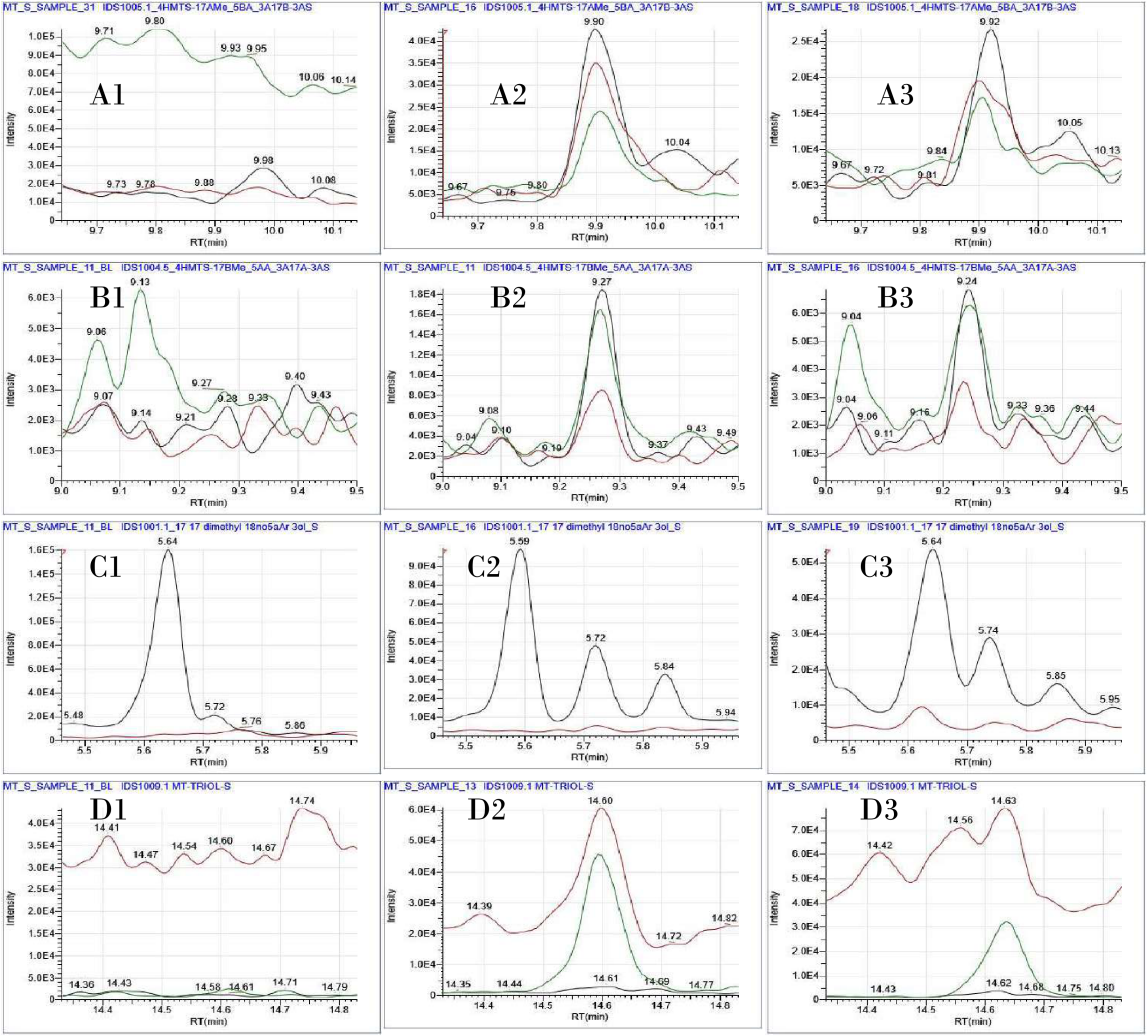
Representative extracted ion chromatograms of TMS derivatives after GC–MS/(MS) analysis of post‐administration samples for (A) M3‐S in a (A1) blank sample, (A2) 8 days post‐administration sample (last identified), and (A3) 11 days post‐administration sample (last detected), (B) M4‐S in a (Β1) blank sample, (Β2) 6 days post‐administration sample (last identified), and (B3) 11 days post‐administration sample (last detected). (C) M6‐S in a (C1) blank sample, (C2) 8 days post‐administration sample (last identified), and (C3) 11 days post‐administration sample (last detected), and (D) M8‐S in a (D1) blank sample, (D2) 8 days post‐administration sample (last identified), and (D3) 10 days post‐administration sample (last detected).

In order to evaluate the developed LC‐HRMS/(MS) method for CDI derivatives detection, and to compare the different available strategies for the detection and identification of the compounds of interest, we repeated the analysis of intact tetrahydro‐methyltestosterone sulfate metabolites in GC‐MS/(MS) after TMS derivatization. The obtained results confirm the findings of Albertdotsir et al. [11]. The GC‐MS/(MS) method enables the identification of a larger number of MT metabolic markers under the provisions of WADA TD2023IDCR, including the M6‐S and M7‐S metabolites, which cannot be identified by the LC‐HRMS/(MS) method after CDI derivatization due to the absence of suitable derivatization sites. Additionally, this approach revealed a previously unreported metabolite coded as M8‐S (Figure 2), which is an isomer of 16z‐hydroxy‐tetrahydro‐methyltestosterone sulfate metabolite detected after full scan analysis of the post‐administration samples. In Table 2 are presented the metabolites with the longest identification time window, along with their RT and their ion transitions at specific collision energies (ce). Representative ion chromatograms for the main intact MT sulfate metabolites detected as TMS derivatives by GC‐MS/(MS) are presented in Figure 7.

**TABLE 2:** Selected tetrahydro‐methyltestosterone metabolites detected as de‐sulphated artifacts after TMS derivatization by GC‐MS/(MS).

Metabolite	RT (min)	Ion transitions/ce (*m/z*/eV)
M3‐S^a^	9.96	270 > 213/ (22) 270 > 228/ (18) 270 > 199/ (28)
M4‐S^b^	9.27	345 > 201/ (20) 270 > 216/ (10) 255 > 189/ (10)
M6‐S^b^	5.75	255 > 161/ (18) 270 > 161/ (24)
M8‐S^b^	14.61	448 > 231/ (9) 448 > 143/ (24) 218 > 147/ (9)

^a^ Detected as multiple peaks.

^b^ Detected as a double peak.

Based on the results, the most suitable sulfate markers for the identification of MT abuse in the frame of sports drug testing, when analyzed as TMS derivatives by GC‐MS/(MS), were M3‐S and M6‐S. Regarding the metabolites M4‐S and M5‐S, which displayed the best detectability when analyzed as intact sulfate metabolite in LC‐HRMS/(MS), they could be identified only in a few, not consecutive, post‐administration samples. This observation was mainly attributed to the very low concentrations of these metabolites, the unfavorable full scan spectra in which the precursor ions for the subsequent PRM analysis represented only a small percentage of the dominant peak at *m/z* 143 [30], and the occurrence of multiple chromatographic peaks corresponding to these de‐sulfated artifacts.

**FIGURE 8:**
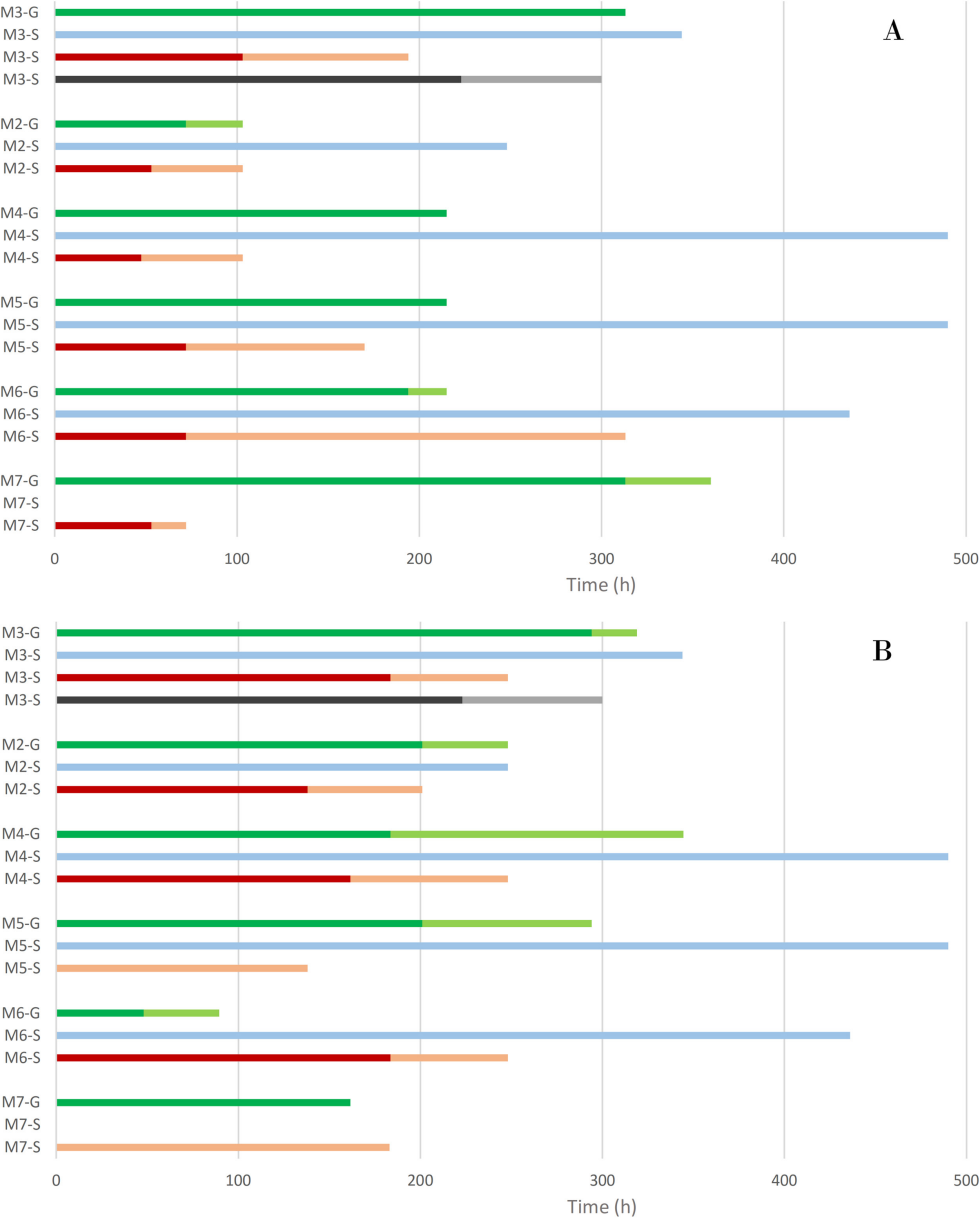
Detection (light lines) and identification (bold lines) time windows for the main MT metabolites of series I (A) and in series II (B) post‐administration samples analyzed by GC‐MS/(MS) as metabolites in the free and glucuronide fractions after hydrolysis (green), LC‐HRMS/(MS) as intact sulphate metabolites (blue), GC‐MS/(MS) as de‐sulfated artifacts of intact sulphate metabolites after TMS derivatization (red) and LC‐HRMS/(MS) after CDI derivatization of intact sulphate metabolites (grey).

The results regarding the detection and identification time windows for the main intact MT sulfate metabolites from both excretion studies I and II are presented in Figure 8A,B, respectively. In summary, while the inclusion of M4‐S in LC‐HRMS initial testing procedure (ITP) can significantly enhance the detection time window for monitoring MT abuse in the context of sports drug testing, its unambiguous identification remains an analytical challenge. The evaluation of the results for MT metabolites excreted as sulfate conjugates in the corresponding confirmation procedures (CP) resulted in similar or shorter identification time windows compared to those obtained from the detection of these metabolites in the free and glucuronide fractions after hydrolysis (e.g., Μ3‐G).

## 4 Conclusion

The microscale synthesis of several tetrahydro‐methyltestosterone sulfate metabolites was performed, and synthetic products were used as standards for the targeted investigation of sulfate metabolites of methyltestosterone in post‐administration samples. This targeted approach enabled the structural elucidation of important tetrahydro‐methyltestosterone metabolites, including the previously reported long‐term metabolites M4‐S and M5‐S, and revealed an additional unreported tetrahydro‐methyltestosterone sulfate metabolite, namely 17α‐methyl‐5α‐androstane‐3β,17β‐diol‐3β‐sulfate (M1‐S). Furthermore, the synthetic standards were used to develop a novel derivatization method for the identification of intact tetrahydro‐methyltestosterone sulfate metabolites by LC‐HRMS, in which product ion spectra could be related to the steroid moiety. The comparison of the identification time windows resulted in time windows that were similar to or shorter than those obtained for the corresponding free and glucuronide counterparts, based on the well‐established GC‐MS/(MS) methods. This finding highlights the moderate effectiveness of current direct identification methods for these metabolites. Further research is required to develop more efficient identification methods that will utilize the extended detection windows offered by including tetrahydro‐methyltestosterone sulfate metabolites, such as M4‐S, in ITPs.

## Author Contributions

**Yiannis S. Angelis:** conceptualization, funding, formal analysis, data curation, manuscript writing, manuscript editing. **Olga Goula:** formal analysis, data curation, manuscript editing. **Polyxeni Kiousi:** formal analysis, data curation, manuscript editing. **Panagiotis Sakellariou:** formal analysis, data curation, manuscript editing.

## Funding

This work was supported by the World Anti‐Doping Agency 16A18IA.

## Conflicts of Interest

The authors declare no conflicts of interest.

## Data Availability

The data that support the findings of this study are available from the corresponding author upon reasonable request.
